# Red propolis and *L*-lysine on angiogenesis and tumor growth in a new model of hamster cheek pouch inoculated with Walker 256 tumor cells

**DOI:** 10.31744/einstein_journal/2019AO4576

**Published:** 2019-04-22

**Authors:** Camila de Carvalho Juanes, Susana Moreira de Souza, Vanessa Nogueira Lages Braga, Francisco Stefânio Barreto, Gisele Rocha Aguiar, Kleison Douglas Gomes Pimentel, Francisco Vagnaldo Fechine, Conceição Aparecida Dornelas

**Affiliations:** 1 Universidade Federal do Ceará, Fortaleza, CE, Brazil

**Keywords:** Neovascularization, physiologic, Lysine, Carcinoma 256, Walker, Cricetinae, Neovascularização fisiológica, Própole, Lisina, Carcinoma 256 de Walker, Cricetinae

## Abstract

**Objective:**

To evaluate the effect of red propolis and *L*-lysine on angiogenesis and tumor growth in a new model of hamster cheek pouch inoculated with Walker 256 tumor cells.

**Methods:**

The study consisted of two experiments with four groups each (total: 57 hamsters). In the experiment 1, the animals were inoculated with Walker tumor cells, followed by administration of test substances (red propolis 200mg/5mL/kg or *L*-lysine 150mg/kg) or control substances (gum arabic 5mL/kg or water 5mL/kg) for 10 days. The animals in the experiment 2 received red propolis, *L*-lysine, gum arabic or water at the same doses, for 33 days prior to inoculation of Walker tumor cells, followed by 10 days of treatment with the same substances. Based on single-plane images, angiogenesis was quantified (mean vascular area), in percentage, and tumor area (mm^2^) and perimeter (mm).

**Results:**

In the experiment 1, compared to animals receiving water, the mean vascular area expressed in percentage was significantly smaller in animal treated with propolis (p<0.05) and *L*-lysine (p<0.001).

**Conclusion:**

Both red propolis and *L*-lysine inhibited tumor angiogenesis in the new hamster cheek pouch model when administered after tumor inoculation.

## RESUMO

**Objetivo:**

Avaliar o efeito da própolis vermelha e da *L*-lisina na angiogênese e no crescimento tumoral em novo modelo de bolsa jugal de hamster inoculada com células de tumor de Walker 256.

**Métodos:**

O estudo consistiu em dois experimentos com quatro grupos cada (total: 57 hamsters). No experimento 1, os animais foram inoculados com células de tumor de Walker, tendo em seguida administradas as substâncias teste (própolis vermelha 200mg/5mL/kg ou *L*-lisina 150mg/kg) ou controle (goma arábica 5mL/kg ou água 5mL/kg) por 10 dias. Os animais do experimento 2 receberam própolis vermelha, *L*-lisina, goma arábica ou água nas mesmas doses, por 33 dias antes do inóculo das células de tumor de Walker, seguido por 10 dias de tratamento com as mesmas substâncias. Baseado em imagens em plano único, foram quantificados a angiogênese (área vascular média), em termos percentuais, e a área (mm^2^) e o perímetro (mm) do tumor.

**Resultados:**

Comparada aos animais que receberam água, a área vascular média, expressa em percentagem, foi significativamente menor nos animais tratados com própolis (p<0,05) e com *L*-lisina (p<0,001).

**Conclusão:**

Tanto a própolis vermelha quanto a *L*-lisina inibiram a angiogênese no novo modelo de bolsa jugal de hamsters, quando administradas após a inoculação do tumor.

## INTRODUCTION

Angiogenesis (or neovascularization) is the formation of new blood vessels from existing ones.^(^ ^1^ ^)^ In 1970, Folkman opened up new perspectives for cancer therapy suggesting that tumor growth was related to and dependent on neovascularization. The ensuing discovery of the first endogenous angiogenesis inhibitors confirmed his hypothesis, and gave rise to a frantic search for new models to study angiogenesis and anti-angiogenic compounds among the molecules known to be present in biodiversity products. In parallel, the pharmaceutical industry developed a number of angiogenesis inhibitors, benefiting thousands of patients. However, due to the complexity of cancer, tumor growth and angiogenesis, many commercially available drugs are only effective against certain types of tumor.^(^ ^2^ ^)^

Brazilian red propolis, a water-insoluble resinous mixture of saliva of bees (*Apis mellifera*) and vegetable exudate, mainly from *Dalbergia ecastaphyllum (L.) Taub*.,^(^ ^3^ ^)^ has strong antioxidant activity and has been investigated and proposed as inhibitor of angiogenesis.^(^ ^4^ ^,^ ^5^ ^)^ *L*-lysine is an essential amino acid which has been shown to promote carcinogenesis^(^ ^6^ ^)^ and to stimulate angiogenesis in induced bladder cancer.^(^ ^7^ ^)^

Walker tumor cells have been used in several tumor implant models.^(^ ^8^ ^)^ The cheek pouch of hamster (*Mesocricetus auratus*)^(^ ^9^ ^,^ ^10^ ^)^ is a tissue suitable for a new model, since the membrane enables visualizing vessels. The hamster cheek pouch implant model described in the literature inoculated tumor fragments, not cells.^(^ ^9^ ^)^ In our experimental model, a standardized number of tumor cells were inoculated to achieve greater consistency of tumor growth between individuals, thus yielding more reliable results.

Several studies have evaluated the immunomodulating activity of propolis. Dornelas et al.,^(^ ^6^ ^)^ observed inhibition of carcinogenesis in animals submitted to 30 days of treatment with propolis, prior to carcinogen inoculation. The role of the immune system in the development of neoplasm is well documented, and the individual immunological profile is known to determine the prognosis of cancer patients. Thus, immunomodulating compounds may be useful in cancer treatment. Complement, lymphocyte and macrophage activation has been observed in many studies, suggesting they are part of the mechanism by which propolis induces apoptosis in tumor cells.^(^ ^11^ ^–^ ^13^ ^)^ In this way, we therefore included an experiment with 33 days of treatment with propolis prior to inoculation (experiment 2).

## OBJECTIVE

To evaluate red propolis and *L*-lysine effect on angiogenesis and tumor growth in a new model of hamster cheek pouch inoculated with Walker 256 tumor cells.

## METHODS

### Study protocol

**Figure 1:**
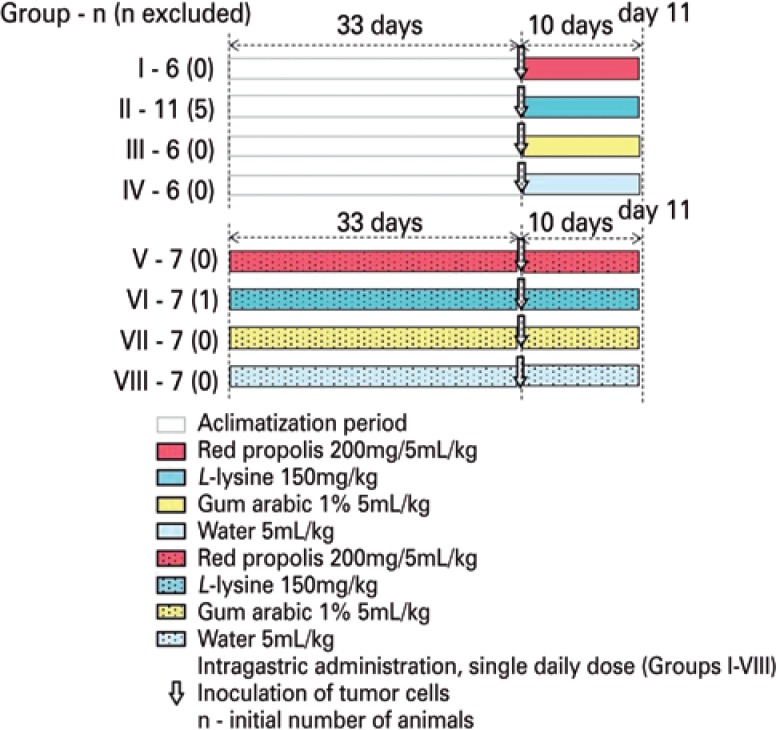
Study design. Experiment 1 included Groups I-IV and experiment 2, Groups V-VIII

The study protocol followed the guidelines of the Brazilian Society of Animal Experimentation and was approved (protocol 89/2015) by the *Comissão de Ética no Uso de Animais* (CEUA). The experiment involved 57 female hamsters (*Mesocricetus auratus*) aged 120 days. The animals were housed in polypropylene boxes, at 25°C, under a 12-hour light/dark cycle, with food and water *ab libitum*. Six animals were excluded, and the final sample had 51 animals. Figure 1 shows the experiment design.

### Preparation and administration of drugs

*L*-lysine monohydrochloride (C_6_H_14_N_2_O_2_·HCl, CAS#657-27-2, FAGRON, China) was diluted in distilled water and administered by oral gavage at a dose of 150mg/kg.^(^ ^6^ ^,^ ^7^ ^)^

Red propolis *in natura* was acquired from a trusted supplier in Barra de Santo Antônio (Alagoas, Brazil) and submitted to extraction in 95% ethanol, at room temperature. After ethanol evaporation, the resulting extract was stored at 4°C, and later diluted in 1% gum Arabic,^(^ ^14^ ^)^ at approximately 60°C, and administered by oral gavage at a dose of 200mg/5mL/kg.

Gum arabic (CAS#9000-01-5, Dinâmica Química Contemporânea LTDA.) was diluted in distilled water solution at 1% and administered by oral gavage at a dose of 5mL/kg.

### Walker 256 tumor cells and inoculation in hamster cheek pouch

**Figure 2:**
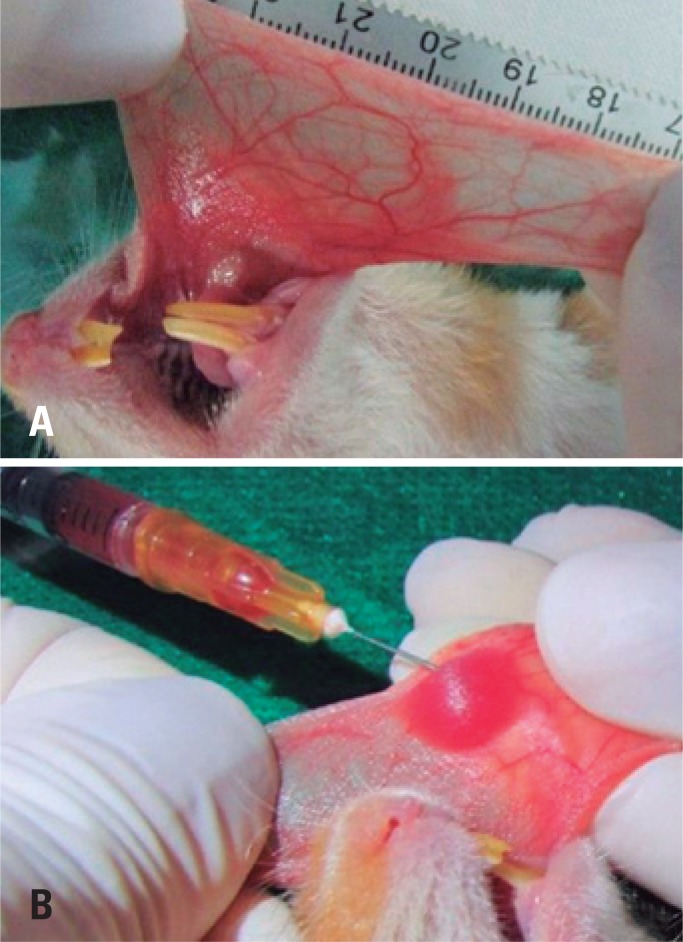
Inoculation of Walker tumor cells. (A) Everted hamster cheek pouch. (B) Inoculation of Walker tumor cells in hamster cheek pouch

The tumor cells were supplied by the National Laboratory of Experimental Oncology. Following anesthesia with an intraperitoneal administration of ketamine hydrochloride (100mg/kg) and xylazine (10mg/kg), the left cheek pouch was everted and washed with saline solution. A 0.1mL-Ringer lactate and gentamicin (50:1) solution containing 1.2×10^6^ Walker tumor cells was injected into the subepithelium, with an insulin syringe and hypodermic needle. To avoid contact with lymph vessels, the inoculum was placed at the center of the cheek pouch, at a safe distance from the retractor muscle fibers (Figure 2).

### Quantification of angiogenesis and tumor size

The tumor was photographed on the 11^th^ day after inoculation. To this end, after the anesthetic procedure described above, the cheek pouch was everted and resected at the base, while cauterizing the blood vessels to prevent drainage of the vessels feeding the tumor. The specimen was then spread out and fastened on a white-bottomed Petri dish. Panoramic (4x, 6x, 10x) and quadrant (16x) micrographs were taken using an analogic video camera (Hitachi VCC-151, Japan), coupled to a stereoscopic microscope (D.F. Vasconcellos S.A., São Paulo, Brazil). The digital images were stored on a notebook running video capture software (PixelView, Prolink Microsystem Corp., Taiwan).

**Figure 3:**
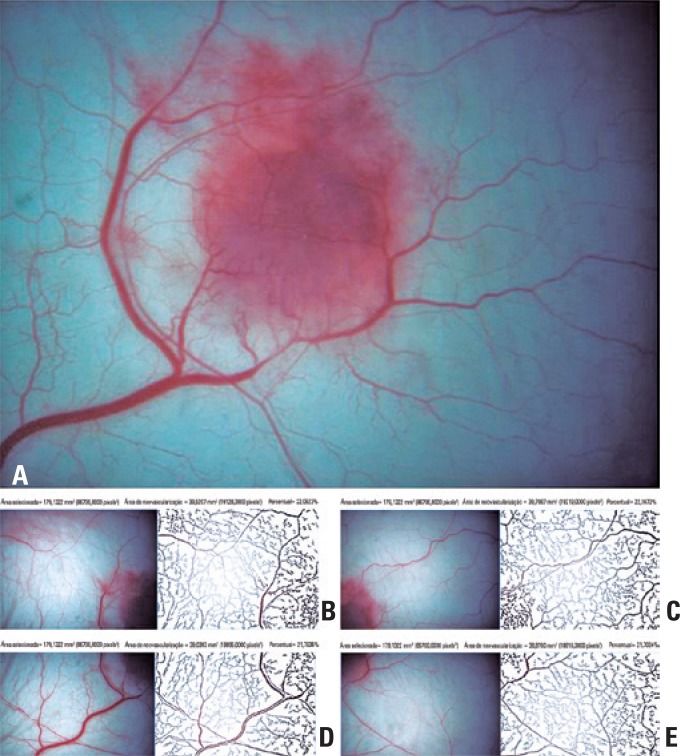
Walker carcinosarcoma implanted in hamster cheek pouch. (A) Panoramic view (10x) of implanted Walker carcinosarcoma in hamster cheek pouch. (B to E) Quadrant view (16x) of vascular area using the System Quantification of Angiogenesis

Angiogenesis was determined by quantifying the quadrant mean vascular area (images 16x), using the System Quantification of Angiogenesis (SQAN)^(^ ^15^ ^)^ (Figure 3). The results were expressed in percentage.

Based on single-plane panoramic images (4x, 6x and 10x), the tumor area (mm^2^) and perimeter (mm) were determined using the software ImageJ (Fiji).^(^ ^16^ ^)^

### Harvesting of organs

The lungs, liver and spleen were excised and weighed, after euthanasia (11^th^ day) with an overdose of anesthetics. In addition, the harvested organs were examined for the presence of metastases.

### Statistical analysis

The normality of distribution of the quantitative variables was verified with the Kolmogorov-Smirnov test. For descriptive statistics, we calculated mean values and standard deviations for all parametric variables. The groups in the experiment 1 (I to IV) and the experiment 2 (V to VIII) were compared pairwise with one-way analysis of variance (ANOVA) associated with Tukey's multiple comparison test. All tests were two-tailed and the level of statistical significance was set at 5% (p<0.05). All analyses were performed with the software GraphPad Prism^®^ version 5.00.

## RESULTS

During the experiment, six animals were excluded due to inadequate inoculation. No metastases were observed in the harvested organs. No animal died during the study period.

The increase in body weight variation was significantly greater in animals treated with *L*-lysine than in animals receiving water (Group II 12.59±6.38% *versus* Group IV 4.03±2.44%; p<0.005; Group VI 32.06±3.30% *versus* Group VIII 22.78±5.41%; p<0.005). As for the harvested organs, the only significant difference was observed for liver in animals treated with *L*-lysine, as compared to liver of animals receiving water (Group VI 7.11±0.92g *versus* Group VIII 5.22*±*1.00g, p<0.005).

In the experiment 1, red propolis (Group I) and *L*-lysine (Group II) significantly reduced the mean vascular area. The groups in the experiment 2 (V to VIII) did not differ significantly in this regard (Table 1).

**Table 1:** Mean vascular area (%) of groups receiving propolis, *L*-lysine, gum arabic, and water for 10 days after inoculation (Groups I to IV) and for 33 days before and 10 days after inoculation (Groups V to VIII)

Mean vascular area (%)	Group I Propolis	Group II *L*-lysine	Group III Gum arabic	Group IV Water	p value (ANOVA)
Mean±SD	22.61±0.6*	22.15±0.42†	22.91±0.53	23.47±0.29	0.0015

Mean vascular area (%)	Group V Propolis	Group VI *L*-lysine	Group VII Gum arabic	Group VIII Water	p value (ANOVA)
Mean±SD	22.58±0.48	22.54±0.34	23.05±0.55	22.83±0.45	0.1958

* p<0.05 and

† p<0.001 indicate significant differences in relation to Group IV (Tukey test). ANOVA: one-way analysis of variance; SD: standard deviation.

No significant difference in tumor area (mm^2^) and perimeter (mm) was observed between the experimental groups (Groups I, II, V and VI) and their respective Control Groups (Tables 2 and 3).

**Table 2:** Tumor area and perimeter of groups receiving propolis, *L*-lysine, gum arabic, and water for 10 days after inoculation

Tumor size	Group I Propolis Mean±SD	Group II *L*-lysine Mean±SD	Group III Gum arabic Mean±SD	Group IV Water Mean±SD	p value (ANOVA)
Area, mm^2^	64.34±42.46	145.18±168.49	120.59±115.52	200.35±59.57	0.2156
Perimeter, mm	69.90±39.57	99.90±43.96	82.12±43.68	97.54±25.77	0.5180

Tukey test. ANOVA: one-way analysis of variance; SD: standard deviation.

**Table 3:** Tumor area and perimeter of groups receiving propolis, *L*-lysine, gum arabic, and water for 33 days before and 10 days after inoculation

Tumor size	Group V Propolis Mean±SD	Group VI *L*-lysine Mean±SD	Group VII Gum arabic Mean±SD	Group VIII Water Mean±SD	p value (ANOVA)
Area, mm^2^	134.25±112.83	80.49±25.34	81.92±26.28	73.13±34.63	0.2713
Perimeter, mm	71.75±30.03	55.62±12.05*	101.00±17.92	68.07±15.02†	0.0035

* p<0.01 and

† p<0.05 indicate significant differences in relation to Group VII (Tukey test). ANOVA: one-way analysis of variance; SD: standard deviation.

## DISCUSSION

The study of neovascularization is important in many fields of pathology, especially cancer progression. In this study, we adapted an experimental hamster pouch model to the study of tumor-induced angiogenesis. Initially, we conducted a pilot study to determine the minimum number of Walker tumor cells required for tumor growth. Care was taken to avoid accidents, such as leaking of tumor cell suspension and inoculation in the vicinity of muscle fibers, and animals with inadequate inoculation (a natural consequence of the learning curve) were excluded from the analysis. The tumor take rate in the remaining animals was 100%.

The electrical cauterization of the blood vessels to prevent drainage and the use of a white background for the capture of digital images through a stereoscopic microscope was a novel method to quantify the angiogenesis in hamster cheek pouch, but since the method required resecting the cheek pouch tissue, angiogenesis could be monitored *in vivo.*

Researchers using the hamster cheek pouch model to study chemical carcinogenesis and neovascularization employed molecular markers and histological sections to quantify angiogenesis,^(^ ^10^ ^)^ but not digital images of fresh specimens taken through a stereoscopic microscope. The software SQAN was designed to quantify angiogenesis in digital images of tissue acquired *in vivo* using a camera coupled to a stereoscopic microscope. Considered quick and practical, the method was used successfully in a rabbit cornea model.^(^ ^15^ ^,^ ^17^ ^)^ This method was validated for morphometric analysis of the vascular network in experimental cancer models.^(^ ^18^ ^)^

Body weight variation was significantly greater in animals treated with *L*-lysine (Groups II and VI) than in animals receiving water. As for the harvested organs, the only significant difference was observed for liver in *L*-lysine group (Group VI) when compared to water group (Group VIII). The body mass gain by *L*-lysine matches the literature.^(^ ^19^ ^)^

A comparison of the mean vascular area, expressed in percentage, in the experiment 1 shows that tumor-induced angiogenesis was only significantly inhibited in animals treated with propolis (Group I) and *L*-lysine (Group II). When submitted to the radical scavenging assay for antioxidant activity, the red propolis used in this study was found to be superior to the vitamin C standard. In addition, the ethanolic extract yielded four isoflavones, one chalcone and one triterpene. The antioxidant properties of red propolis were related to the ability of chalcones and isoflavonoids to donate electrons.^(^ ^20^ ^)^ The total phenolic content in the ethanolic extract was 133.3±4.35mg GAE/g of sample.^(^ ^21^ ^)^ The levels of antioxidant activity and phenolic compounds observed in this study match those of previous analyses.^(^ ^5^ ^)^ These findings justify the study of red propolis extract as a potential anti-angiogenic agent. This antioxidant action may act both hindering the tumor development as well as with the inhibition of angiogenesis, since oxidative stress underlies of the pathophysiology of cancer.^(^ ^22^ ^)^

Red propolis produces antioxidant effects, inhibits angiogenesis through modulation of angiogenic factors and inflammation, and reduces the levels of vascular endothelial growth factor (VEGF) and hypoxia-inducible factor (HIF)-1α.^(^ ^23^ ^,^ ^24^ ^)^ Also extensively documented is the relation between angiogenesis, oxidative stress and tumor hypoxia.^(^ ^25^ ^)^ The correlation between the antiangiogenic and antioxidant effects of propolis was evaluated *in vitro* using endothelial cells; unsurprisingly, the most antiangiogenic compounds were also the most antioxidant.^(^ ^26^ ^)^

Few studies have been conducted on the effect of *L*-lysine on angiogenesis. In one study, *L*-lysine promoted angiogenesis in chemically induced bladder cancer, when administered concomitantly with the carcinogen, leading the authors to rise the hypothesis that angiogenesis may have contributed to tumor size and aggressiveness.^(^ ^7^ ^)^

Interestingly, the vascular area decreased very slightly in animals receiving red propolis and *L*-lysine for 43 days (pre + post inoculation of Walker tumor cells), in experiment 2, compared to the Control Groups receiving water. Angiogenesis was not inhibited as in animals treated with propolis and *L*-lysine for 10 days (experiment 1). This finding is not easy to explain, but different studies have found cytotoxic or cytoprotective effects depending on the cells being neoplastic or normal, and on the experiment being *in vitro* or *in vivo*. Even antioxidant and oxidant activity may be related to the dose and time of administration. Therefore antagonistic effects can be explained by the different compounds present in the propolis product, as well as the narrow threshold between therapeutic and toxic dose.^(^ ^27^ ^,^ ^28^ ^)^

Whether red propolis and *L*-lysine are suitable as adjuvants to other angiogenesis inhibitors remains to be confirmed. So far, even angiogenesis receptor inhibitors are inefficient against many tumor types, either because tumor growth factor receptors differ, or because angiogenesis is dependent on non-VEGF pathways, as in lung cancer. It remains to be determined whether propolis is effective in non-neoplastic conditions, such as psoriasis and endometriosis.^(^ ^29^ ^)^

No significant difference in tumor area and perimeter was observed between the experimental groups and their respective Control Groups. However, single-plane measurements only reflect the surface of the tumor, not taking into account the entire tumor volume.

Red propolis and isolated compounds modulate the progression of carcinogenesis *in vivo*,^(^ ^30^ ^)^ and are cytotoxic to lineages of tumor cells *in vitro.* ^(^ ^27^ ^)^ However, a study with tumor cell lineages revealed that different concentrations of red propolis are associated with different profiles of cytotoxicity.^(^ ^27^ ^)^

*L*-lysine, in turn, when administered concomitantly with the carcinogen, promoted carcinogenesis in chemically-induced bladder cancer, and protected against genotoxicity in bone marrow and peripheral blood. Nonetheless, animals receiving *L*-lysine alone did not develop cancer. Hence, although *L*-lysine has a (non-genotoxic) promoter action on bladder carcinogenesis, is not genotoxic to leukocytes from bone marrow or peripheral blood at doses tested in animals.^(^ ^6^ ^,^ ^31^ ^)^

On the other hand, *L*-lysine failed to promote carcinogenesis in a rat model of bladder cancer submitted to ureterosigmoidostomy and vesico-sigmoidostomy.^(^ ^32^ ^,^ ^33^ ^)^ However, *L*-lysine accelerated the development of transitional metaplasia in intestinal epithelium in rats submitted to cystoplasty.^(^ ^34^ ^)^

## CONCLUSION

Both red propolis and *L*-lysine inhibited angiogenesis in new model hamster cheek pouch when administered after tumor inoculation.
